# Enhancing Perinatal Safety with the Advancement of Obstetric Anesthesia in Japan

**DOI:** 10.1089/whr.2024.0154

**Published:** 2025-01-13

**Authors:** Reiko Ohara

**Affiliations:** Department of Anesthesia, National Center for Child Health and Development, Tokyo, Japan.

**Keywords:** labor analgesia, obstetric anesthesia, safety advancement, Japanese perinatal system;

## Abstract

Japan is one of the most developed countries in the world, and perinatal care is safe, with low maternal and neonatal mortality rates. However, as birthrate declines, advanced maternal age and the number of cesarean deliveries increases, efforts must be made to maintain safety in the future. The characteristic of the delivery facilities is “many small clinics,” and half of all facilities have fewer than 500 deliveries per year. Such clinics rarely have full-time anesthesiologists; therefore, anesthetic management, including cesarean deliveries and labor analgesia, is left to obstetricians’ efforts. Furthermore, the prevalence of labor analgesia is very low compared with that in developed countries. In 2017, maternal deaths during labor analgesia were reported and became a social problem. The Ministry of Health, Labor, and Welfare (MHLW) appointed a research group to conduct a study on labor analgesia and issued recommendations for a safe delivery system in 2018. Even when recommendations are understood, changing the size and staffing of medical facilities is challenging, and the field needs specific interventions such as education and information sharing. Most maternal deaths related to anesthesia are preventable. Within the existing medical environment, a distinctive anesthetic management system is crucial to improve the safety of perinatal care, and cooperation among obstetricians, anesthesiologists, nurses, and midwives involved in perinatal care through education and management is essential. We review past perinatal safety initiatives in Japan and discuss the need to make obstetric anesthesia safer as future risks increase.

## Background

**FIG. 1.**
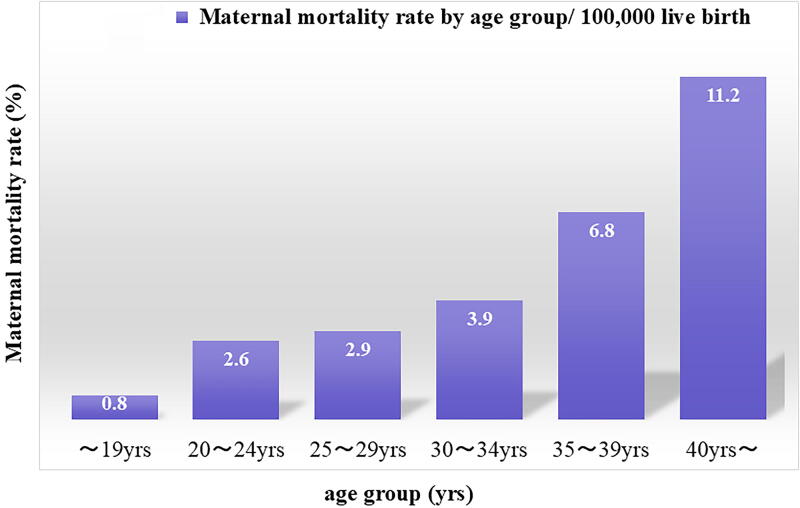
It shows Japanese maternal mortality rates by age group; the data were examined for all maternal deaths in Japan for an 11-year period, from 2010 to 2021. Pregnant women over 40 years of age had significantly higher mortality rates compared to other age groups (*P* < 0.01). Source: High maternal mortality rate associated with advanced maternal age in Japan. Scientific reports.2023;13:12918.

According to the Ministry of Health, Labor and Welfare (MHLW), the number of births in Japan declined for the 8th consecutive year, falling below the 800,000 level to 758,631 in 2023.^1^ In terms of maternal mortality, statistics from 2010 to 2021 show that the maternal mortality rate for those aged 40 and older is 11.2/100,000 live births, four times the rate for those in their 20s, indicating that the risk increases with childbearing age (Fig. 1).^2^

Perinatal care in Japan has a characteristic delivery facility due to a lack of centralized delivery facilities and many “small delivery clinics,” which manage half of all deliveries.^3^ These clinics have limited medical equipment and specialized personnel and must transport patients to higher-level medical facilities in an emergency. Few anesthesiologists were involved in obstetric anesthesia at any of these small delivery clinics.

To make perinatal care in Japan safer from the standpoint of anesthesiologists, sharing past efforts and problems in perinatal care and future possibilities for safe obstetric anesthesia in Japan.

## The Progress of Safety in Perinatal Care in Japan

Since 1980, Japan’s perinatal care for premature, low-birthweight, and sick newborns have made remarkable progress. In 1996, the government established a system to provide 24-hour emergency care for expectant mothers and newborns in the community. By 2011, all prefectures had designated comprehensive and regional perinatal maternal and newborn centers to ensure smooth transportation of mothers and newborns. As a result, maternal and newborn transport has eased in each region, and adverse birth outcomes have become extremely rare.

**FIG. 2.**
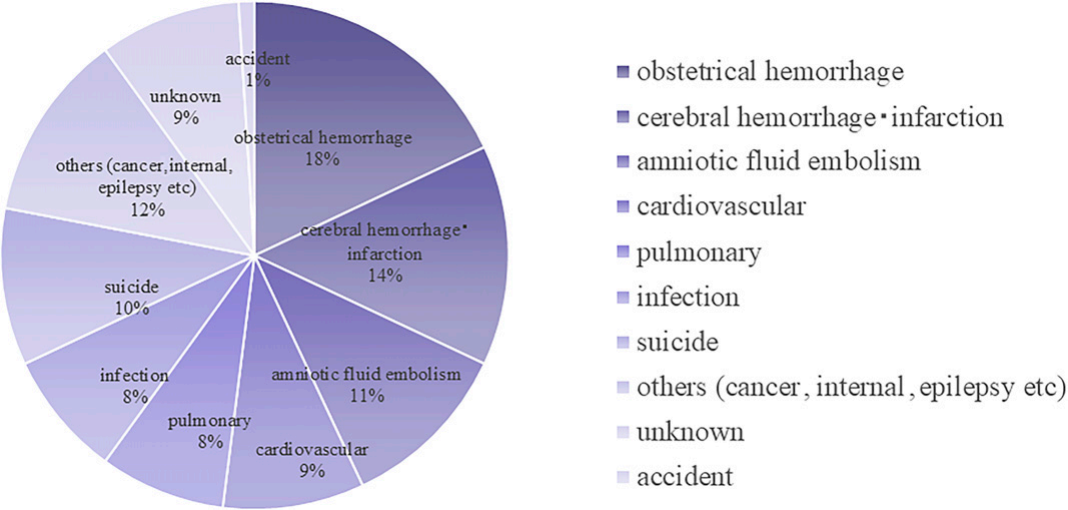
It shows that the most common cause of maternal mortality over the 13 years from 2010 to 2022. Obstetric hemorrhage accounted for 18% of cases and was the most common cause of maternal mortality. This was followed by intracranial hemorrhage or infarction (14%), amniotic fluid embolism with cardiopulmonary collapse (11%), perinatal myocardiopathy (9%), and pulmonary thromboembolism (9%). Cardiovascular disease (a combination of cardiac diseases such as peripartum cardiomyopathy and aortic dissection), pulmonary diseases, such as pulmonary thromboembolism, infectious diseases (*e.g.,* fulminant group A streptococcal infection) accounted for 8%, and suicide accounted for 10%. Source: The Japan Maternal Death Explorating Committee 2022 of the Japan Association of Obstetricians and Gynecologists. 2023 September.

The maternal mortality rate has decreased from 176.1 in 1950 (per 100,000 live births) to 4.2 in 2022. The perinatal mortality rate decreased from 20.2 in 1980 (per 1,000 live births) to 3.2 by 2022.^1^ The Japan Association of Obstetricians and Gynecologists (JAOG) conducted a cause-of-death analysis of maternal mortality, which reported maternal deaths from 2010 to 2022, and allowed for the cause-of-death analysis (Fig. 2). The results showed that the leading cause of maternal death was postpartum hemorrhage, the second leading cause was cerebral hemorrhage, and the third leading cause was amniotic fluid embolism. A detailed investigation of the causes of hemorrhage and death revealed that amniotic fluid embolism was the most common cause, with uterine rupture, placental abruption, adhesive placenta, and atonic hemorrhage being the other most common.^4^

Blood transfusion is usually unavailable in small maternity facilities, and there are few medical personnel, making it challenging to manage hemorrhage during labor and delivery. To address this issue, the JAOG, the Japan Society of Obstetrics and Gynecology (JSOG), the Japan Society of Perinatal and Neonatal Medicine, the Japanese Society of Anesthesiologists (JSA), and the Japan Society of Transfusion Medicine and Cell Therapy developed “Guidelines for the Management of Critical Obstetric Bleeding” in 2010. This guideline recommends the use of two original indicators: a “bleeding management flow chart” and a “shock index (SI),” as SI expresses heart rate/blood pressure (SI: HR/BP). An SI of 1 indicates an estimated bleeding volume of 1,500 mL, while an SI of 1.5 indicates an estimated volume of 2,500 mL, which makes it easy for obstetricians, nurses, and midwives to quickly estimate the amount of blood loss. SI is beneficial for small medical facilities as it enables them to determine the right time to transfer to higher-level medical facilities. The guidelines have been revised, and in 2022, a description of cases requiring interventional radiology and fibrinogen products has been added.^5^

In addition, the simulation courses were started to prepare for obstetric crises such as hemorrhage were launched in July 2015: The Japan Council for Implementation of Maternal Emergency Life-Saving System was established to disseminate standard maternal life support methods to obstetricians, emergency physicians, anesthesiologists, midwives, nurses, and other professionals involved in perinatal care, and to promote the development and implementation of effective maternal life support systems, thereby contributing to the welfare of society by providing high-quality medical care to parturient and improving perinatal care by providing simulation opportunities. The main activity throughout Japan was to conduct maternal life support training.

Thus, the use of SI along with flow charts to avoid delaying the timing of transportation to higher facilities and simulation training has increased the safety of maternal outcomes.

## Cesarean Delivery and Anesthetic Management

**FIG. 3.**
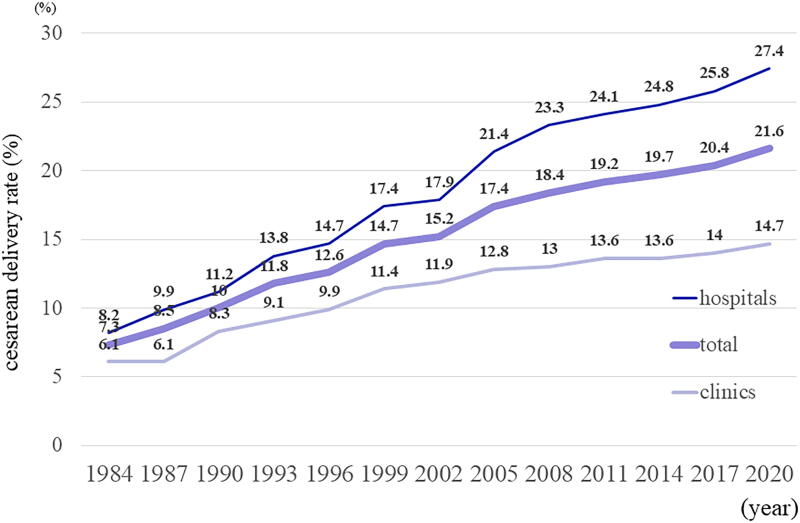
The following are the annual trends in the Cesarean delivery rate as reported by the Medical Static Survey of the Ministry of Health, Labor, and Welfare. There has been a yearly increase in the cesarean delivery rate in both clinics and hospitals. Source: Summary of Vital Statistics for 2020, Ministry of Health, Labor and Welfare, Medical Facilities (static and dynamic) Survey, Summary of Hospital Reports 2020.

Regarding the annual cesarean delivery rate in Japan, the latest report is for 2020, and the cesarean delivery rate was 27.4% for hospital deliveries, 14.7% for clinic deliveries, and 21.6% for all deliveries (Fig. 3).^6^ It was 10% of all deliveries in 1990, but it more than doubled to 21.6% in 2020 and continues to increase annually. According to the survey of deliveries reported by the JAOG, in terms of anesthesia responsibility for cesarean sections, full-time anesthesiologists provide anesthesia for scheduled cesarean sections in 54% of the mean values in hospitals, compared with 3% in clinics, and obstetricians who also serve as surgeons provide anesthesia in 65% of the mean values in clinics. Thus, obstetric surgeons in the clinic are involved in surgery and anesthesia management (Table 1).^3^ Even in hospitals larger than the clinic, full-time anesthesiologists manage only two-thirds of cesarean deliveries, which means that obstetric surgeons manage both anesthesia and surgery.

**Table 1.** In Charge of Anesthesia for Cesarean Delivery

			Mean(SD)
	**Hospital**	**Clinic**	*p* value
Planned cesarean delivery			
ft-AN	54 ± 44%	3 ± 17%	<0.05
pt-AN	14 ± 28%	11 ± 30%	ns
OB	6 ± 21%	13 ± 31%	ns
so-OB	23 ± 40%	65 ± 45%	<0.05
Emergency cesarean delivery			
ft-AN	52 ± 44%	3 ± 17%	<0.05
pt-AN	11 ± 24%	7 ± 23%	ns
OB	6 ± 21%	13 ± 31%	ns
so-OB	27 ± 41%	67 ± 44%	<0.05

ft-AN, full-time anesthesiologist; pt-AN, part-time anesthesiologist; OB, obstetrician; so-OB, surgical operator obstetrician; ns, not significant.

## Labor Analgesia in Japan

In the Western world, “pain” is regarded as a harmful symptom and a subject of medical treatment, and there is a mutual understanding. In the United States, for example, the American College of Obstetricians and Gynecologists has stated the choice of anesthesia for childbirth; in the absence of a medical contraindication, maternal request is a sufficient medical indication for pain relief during labor.^7^

**FIG. 4.**
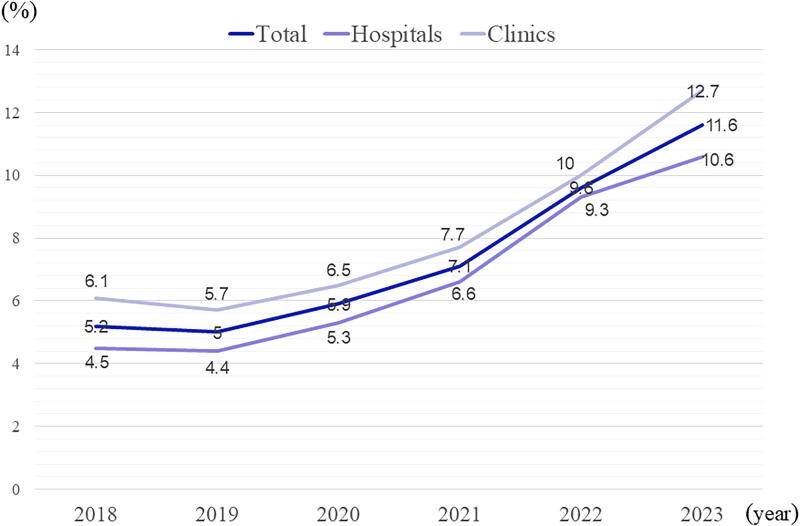
This figure represents the annual percentage of labor analgesia in all deliveries in Japan, which finally exceeded 10% in 2023. Source: Labor analgesia from the standpoint of obstetric facilities: Analysis from the Japan Association of Obstetricians and Gynecologists Facility Information. Yearly Trends in the Percentage of Labor Analgesia to the Total Number of Deliveries.

The history of labor analgesia in Japan is considerably different from that of the West; although the prevalence of labor analgesia in Japan is increasing, it is low, unlike other high-resourced countries, with only 11.6% of all deliveries having labor analgesia in 2023, as shown (Fig. 4).^8^ In 1961, Japanese obstetricians founded a study group on anesthesia for labor and delivery, the “Society for the Study of Labor Analgesia,” the predecessor of the current Japan Society for Obstetric Anesthesia and Perinatology (JSOAP); however, the Japanese traditional view of labor pain, in which the suffering of childbirth is accepted, became dominant. In addition, the Japanese are unfamiliar with labor analgesia partly because there are very few facilities that provide labor analgesia, even if desired.^9^ Concerning Japanese delivery facilities, almost half of the deliveries are managed at clinics by one or two obstetricians, and it is rare to have full-time anesthesiologists or perinatologists.

Under these circumstances, serious maternal and neonatal complications during labor analgesia were reported in a series of news reports in 2017. These included three maternal deaths and two fetal/neonatal deaths that occurred due to total spinal and epidural analgesia for labor at the clinic that had occurred over the previous 10 years (Table 2).^10^ The repeated media coverage has caused enormous public concern about the safety of labor analgesia. Until then, there were no Japanese standards to ensure the safety of labor analgesia; therefore, each facility had developed its system based on its own methods and ideas.

**Table 2.** Severe Maternal and Neonatal Complications during Labor Analgesia

Month/year	Facility	Anesthesia for delivery style	Cause	Maternal prognosis	Child prognosis	Report to medical society
12, 2008	A clinic	epidural for labor	Total spinal	death	death	unknown
04, 2011	B clinic	epidural for labor	unknown	unknown	death after cerebral palsy	incidental unreported
11, 2012	B clinic	epidural for labor	Total spinal	bedridden	cerebral palsy	incidental unreported
02, 2015	C hospital	epidural for labor	Uterine rupture	hysterectomy	death	unknown
09, 2015	D clinic	epidural for labor	Total spinal	death	death	incidental unreported
05, 2016	B clinic	epidural for cesarean delivery	unknown	bedridden	cerebral palsy	incidental unreported
01, 2017	E clinic	epidural for labor	Total spinal	death	healthy	Nurse reported

To build a consensus on standards for safe practices in labor analgesia and to establish a roadmap for the sustainable development of safer systems, the MHLW study group’s investigation was conducted by members of seven Japanese societies related to perinatal care: Japan Medical Association, Japanese Nursing Association, the JSOG, the JAOG, Japan Society of Perinatal and Neonatal Medicine, the JSA, and the JSOAP. In a study of clinical problems and the development of safety measures for labor analgesia, the period between August 2017 and March 2018. From an analysis of the content of reported cases of serious adverse events during labor analgesia, four cases of maternal death were reported. The leading causes of death were epidural analgesia and high spinal anesthesia. Data on the physicians responsible for labor analgesia were most recently reported in 2017, and obstetricians were primarily responsible for labor analgesia. Specifically, in clinics, obstetricians were responsible for 84.9% of all labor analgesia, while anesthesiologists were responsible for only 9.1% (Table 3).^3^

**Table 3.** In Charge of Managing Labor Analgesia in Japan (%)

	Hospital	Clinic	*p* value
obstetrician	62.7%	84.9%	<0.001
trained obstetrician	7.4%	12.9%	0.056
Anesthesiologist	47.0%	9.1%	<0.001

In March 2018, Dr. Nobuya Unno and colleagues, appointed by the MHLW, developed a public agreement, “Recommendations for the Establishment of a System for the Safe Provision of Labor Analgesia.” Specifically, they recommend the following: (1) clarifying medical responsibilities, (2) ensuring technical standards of medical personnel, (3) establishing necessary facilities and medical equipment, (4) medical staff in charge sharing recognition and working as a team, (5) providing sufficient explanations about labor analgesia to parturient.^11^ To realize “the recommendations,” the Japanese Association for Labor Analgesia (JALA) was established in July 2018. The JALA consists of three divisions to enhance Japanese perinatal safety: safety training, disclosure of information, and adverse events analysis. In April 2018, the JALA issued a notice “On the establishment of a safe provision system for labor analgesia” through the MHLW, which means that the “recommendations” and the “voluntary inspection list” based on them will be used as reference materials during on-site inspections by prefectural governments, and “advice” may be provided based on them. In addition, the JALA has provided information on the MHLW website, including the provision of the “List of Facilities Handling Labor Analgesia,” and has been able to enact the addition of a report on labor analgesia to the survey of the static conditions of medical facilities conducted every 3 years to ascertain the actual status of medical treatment, starting in 2020. In this way, the JALA has clarified the needs in the field of labor analgesia and provided specific measures necessary for the medical procedure system, as shown on the website (https://www.jalasite.org/). However, while about 700 institutions provided labor analgesia, 537 institutions have agreed to participate in the JALA project, and 323 institutions were listed on the list, which has gradually increased since the JALA website was launched in 2019.

## History of Advanced Obstetric Anesthesia: An Overview in the United States

With efforts and progress, the status of women in any civilization is an indicator of the care they receive during childbirth. Moreover, the most recent surveys of obstetric anesthesia in countries where obstetric anesthesia is well established report improvements decade by decade basis.^11^ In the United States, where obstetric anesthesia is more advanced, the American College of Obstetricians and Gynecologists (ACOG) and the American Society of Anesthesiologists (ASA) have issued a joint statement that obstetric anesthesia should be provided by anesthesiologists and improvements have been made in this area. In 2007, the ACOG and the ASA issued a joint statement calling for an increase in the proportion of anesthesiologists in the perinatal period, reviewing the evidence for the role of anesthesiologists in reducing maternal mortality and morbidity and making recommendations for anesthesiologists to contribute to these efforts at the local, regional, and national levels.^12,13^

In 1981, obstetricians performed 30% of obstetric anesthesia cases in the United States; by 1992, obstetricians performed less than 6% of cases.^15^

The impetus for this was the 1982 Professional Standards of the American College of Obstetricians and Gynecologists, which recommended that obstetricians should not administer anesthesia, but should entrust it to anesthesiologists specializing in obstetric anesthesia.^15^ What is even more impressive is that Dr. Richard Schmidt, president of ACOG then, began supporting the establishment of the Cincinnati School of Medicine in 1970 to provide anesthesia training and to train obstetric anesthesiologists. His contribution is reported as an explanation for some of the changes noted in the obstetric anesthesia workforce surveys and as an essential step in the development of anesthesiology, especially in obstetric anesthesia.^15^ Many factors influence the number and type of anesthesiologists available and the services they provide. The most recent survey on obstetric anesthesia found that although the availability of regional analgesia and anesthesia for labor and delivery has improved over the past decade, staffing patterns and the availability of human resources still need to be improved. Currently, 66% of deliveries occur in Hospital Group I, which performs more than 1,500 deliveries annually and is a training facility for medical residents.^16^

## Cause of Death during Labor Analgesia

The Japanese perinatal period has endeavored to be safe; however, with the increase in the numbers of complicated pregnancies and cesarean deliveries, increased involvement of anesthesiologists is required. Furthermore, obstetric anesthesia is a medical intervention that should not be a cause of death.

Most cause of death during labor analgesia; the incidence of high spinal cases becoming necessary for airway and circulatory management was reported to be between 1 in 1,400 and 16,200.^17,18^ High spinal anesthesia is an infrequent complication, and it is difficult to prevent its occurrence completely; however, emergent and appropriate treatment may reduce the chances of its progressing to life-threatening complications. Another report by the SOAP described serious complications related to obstetric anesthesia. Thirty institutions took part in the 5-year study period, and of the 145,550 cases of epidural anesthesia, 157 were serious complications, 85 of which were anesthesia-related. High neuraxial block, respiratory arrest during labor and delivery, and unrecognized spinal catheters were the most frequent complications. They have also recommended that anesthesia providers remain vigilant and diagnose and treat rapidly.^19^ Regarding medicolegal issues in obstetric anesthesia in the United States, one report reviewed 106 closed obstetric anesthesia cases between 2005 and 2015. The most common causes of maternal death and brain injury are high-level neuraxial block, embolic events, and failed intubation. High-level neuraxial block during obstetric anesthesia requires prompt diagnosis and treatment with adequate resuscitation resources, including advanced airway management techniques.^20^ It is time to recognize that it is not easy for obstetricians to cope with such a critical situation to provide anesthesia while providing obstetric care, as is the current situation in Japan. The perinatal status in Japan shows that the causes of maternal death include postpartum hemorrhage, embolism, and cerebrovascular and hypertensive disorders, which require early intervention by medical teams before they lead to fatal situations. Furthermore, a previous Japanese study reported that the preventable maternal mortality rate was 14 times lower in referral facilities than in transferring facilities.^21^ It is conceivable that the risk of delivery will increase in the future as the number of advanced maternal ages increases over time.^2^ Considering that obstetricians are responsible for the anesthetic management of cesarean delivery or labor analgesia in Japan, simultaneous obstetrical and anesthetic management by one physician may have unacceptable risks. Support through the education of nurses and midwives, and efforts should be made to involve anesthesiologists safely. It is necessary to educate anesthesiologists who are competent in obstetric anesthesia.

## Further Improvement of Obstetric Anesthesia

To further improve obstetric anesthesia in perinatal care, the SOAP Center of Excellence designation was created in 2018 to recognize institutions and programs that demonstrate excellence in obstetric anesthesia care and to set a benchmark of expected care to improve standards nationally and international standards. It has accredited 83 facilities by 2022. Three Japanese institutions (Juntendo University Hospital, Kitasato University Hospital, and the National Center for Child Health and Development) have been designated as centers of excellence by the SOAP, and some Japanese anesthesiologists have taken the following steps to simultaneously educate and promote obstetric anesthesiologists, although there are few educational institutions.

Furthermore, it has been reported that neuraxial labor analgesia may be associated with reduced odds of maternal blood transfusion in intrapartum cesarean deliveries and, to a lesser extent, vaginal deliveries. In light of this report, possible mechanisms may include sustained monitoring of the parturient during labor analgesia, improved early detection of immediate blood loss, adequate intravenous access to ensure cardiovascular circulation, and continuous anesthesia provider availability and preparedness for acute events. The use of labor analgesia could be a proxy for the provision of high-quality obstetric care.^22^ There is another report that showed that epidural analgesia during labor was associated with a 35% reduction in severe maternal morbidity; the mechanism is likely multifaceted, involving closer medical supervision and hemodynamic monitoring, established intravenous access, fluid administration, blunting of physiological stress responses to labor, avoidance of the need for spinal or general anesthesia for cesarean delivery, and faster escalation to definitive obstetric interventions.^23^ Labor analgesia alters the care pathway and vigilance to enhance the ability to manage adverse events during labor.

## Discussion

Over time, the number of cases of advanced maternal age has increased, leading to higher risks during labor and delivery in Japan. It is essential to reconsider the practice of obstetric anesthesia during labor and delivery, especially in the context of an inadequate medical system. The involvement of trained nurses and midwives educated in obstetric anesthesia is crucial in providing comprehensive care during delivery. Similarly, the involvement of anesthesiologists, especially those educated in obstetric anesthesia, will ensure safe administration of anesthesia during these critical procedures.

A challenge for perinatal care in Japan is the potential for improvement. By rethinking obstetric anesthesia education and the system, we can enhance multidisciplinary team care and improve perinatal care.

## Conclusion

It is no exaggeration to state that the quality of women’s childbirth care is an indicator of the true wealth and progress of a country. Japan is on the brink of a critical period in perinatal care, with a looming threat of increased risk due to the rise in complicated pregnancies from an advanced maternal age. The time to act is now. Obstetric anesthesia requires immediate attention and rethinking.

To improve the safety of perinatal care, anesthesiologists, with their understanding of the perinatal system, are crucial for the improvement of obstetric anesthesia. Involvement and collaboration with other obstetric professionals during the perinatal period are essential to ensure a safe system in the 21st century.

## Data Access Statement

All relevant data are included in the article and its supporting information files.

## References

[1] Reports of the Ministry of Health, Labor and Welfare. Summary of Vital Statistics Monthly Report Annual Total (approximate numbers). 2023. Available from: https://www.mhlw.go.jp/toukei/saikin/hw/jinkou/geppo/nengai23/dl/gaikyouR5.pdf [Last accessed: October 8, 2024].

[2] Tanaka H, Hasegawa J, Katsuragi S, et al. High maternal mortality rate associated with advanced maternal age in Japan. Sci Rep 2023;13(1):12918; doi: 10.1038/s41598-023-40150-4 37558813 PMC10412567

[3] Japan Association of Obstetrics and Gynecology, Reports of the Medical Safety Committee. “Survey of delivery 2017” analysis update March 29. 2023. Available from: https://www.jaog.or.jp/wp/wp-content/uploads/2017/12/20171213_2ver.2.pdf [Last accessed: October 8, 2024].

[4] Maternal Mortality Case Review and Evaluation Committee, Japan Obstetrics and Gynecology Association. 2023. Available from: https://www.jaog.or.jp/wp/wp-content/uploads/2023/01/botai_2022.pdf [Last accessed: October 8, 2024].

[5] Japan Society of Obstetrics and Gynecology. Guidelines for Obstetric Critical Hemorrhage 2022. 2022. Available from: https://www.jsog.or.jp/activity/pdf/shusanki_taioushishin2022.pdf [Last accessed: October 8, 2024].

[6] Summary of Vital Statistics for 2020, Ministry of Health, Labor and Welfare. Medical Facilities (static and dynamic) Survey, Summary of Hospital Reports. 2020. Available from: https://www.mhlw.go.jp/toukei/saikin/hw/iryosd/20/dl/09gaikyo02.pdf [Last accessed: October 8, 2024].

[7] The American College of Obstetricians and Gynecologists. ACOG Practice Bulletin No.209: Obstetric Analgesia and Anesthesia. Obstet Gynecol 2019;133(3):e208–e225. https://journals.lww.com/greenjournal/fulltext/2019/03000/acog_practice_bulletin_no__209__obstetric.45.aspx 30801474 10.1097/AOG.0000000000003132

[8] Matsuda H. Labor analgesia from the standpoint of obstetric facilities: Analysis from the Japan Society of Obstetricians and Gynecologists Facility Information. Yearly Trends in the Percentage of Labor Analgesia to the Total Number of Deliveries. Available from: https://www.jaog.or.jp/wp/wp-content/uploads/2023/09/e553496982d83ca62076fb6974c445b2.pdf [Last accessed: October 8, 2024].

[9] Maeda Y, Takahashi K, Yamamoto K, et al. Factors affecting the provision of analgesia during childbirth, Japan. Bull World Health Organ 2019;97(9):631–636. 31474776 10.2471/BLT.19.230128 PMC6705500

[10] Unno N, Itakura A. Research on the actual situation of labor analgesia and the establishment of the safety management system. Research Project of The Ministry of Health, Labor and Welfare; 2017. Available from: https://mhlw-grants.niph.go.jp/system/files/2017/171031/201706027A_upload/201706027A0007.pdf

[11] Unno N. Research on the actual situation of labor analgesia and the establishment system. March 2019, Ministry of Health, Labor and Welfare Special Scientific Research Project. Proposal for the establishment of a safe delivery system for labor analgesia. Ministry of Health, Labour and Welfare, Special Scientific Research Projects. Available from: https://www.mhlw.go.jp/file/05-Shingikai-12601000-Seisakutoukatsukan-Sanjikanshitsu_Shakaihoshoutantou/0000203226.pdf [Last accessed: October 8, 2024].

[12] Bucklin BA, Hawkins JL, Anderson JR, et al. Obstetric anesthesia workforce survey twenty-year update. Anesthesiology 2005;103(3):645–653. 16129992 10.1097/00000542-200509000-00030

[13] Statement on Optimal Goals for Anesthesia Care in Obstetrics. Committee on Obstetrics and Anesthesia. October 17, 2021. Available from: https://www.asahq.org/standards-and-practice-parameters/statement-on-optimal-goals-for-anesthesia-care-in-obstetrics [Last accessed: October 8, 2024].

[14] American Society of Anesthesiologists. Statement on Anesthesiologists’ Role in Reducing Maternal Mortality and Severe Maternal Morbidity. October 26, 2022. Available from: https://www.asahq.org/standards-and-practice-parameters/statement-on-anesthesiologists-role-in-reducing-maternal-mortality-and-severe-maternal-morbidity [Last accessed: October 8, 2024].

[15] Johnstone RE. 2nd., Obstetric Anesthesia: The 1982 American College of Obstetricians and Gynecologists Standards and the role of Robert E. Johnstones, M.D. Anesthesiology 2006;104(5):1103–1104. 16645464 10.1097/00000542-200605000-00028

[16] Traynor AJ, Aragon M, Ghosh D, et al. Obstetric anesthesia workforce survey: A 30-year update. Anesth Analg 2016;122(6):1939–1946. 27088993 10.1213/ANE.0000000000001204

[17] Paech MJ, Godkin R, Webster S. Complications of obstetric epidural analgesia and anesthesia: A prospective analysis of 10,995 cases. Int J Obstet Anesth 1998;7(1):5–11. 15321239 10.1016/s0959-289x(98)80021-6

[18] Jenkins JG. Some immediate serious complications of obstetric epidural analgesia and anesthesia: A prospective study of 145,550 epidurals. Int J Obstet Anesth 2005;14(1):37–42. 15627537 10.1016/j.ijoa.2004.07.009

[19] Robert DA, Richard MS, Edward TR, et al. Serious complications related to obstetric anesthesia, the serious complication repository project of the society for obstetric Anesthesia and Perinatology. Anesthesiology 2014;120:1505–1512. 24845921 10.1097/ALN.0000000000000253

[20] Vesela PK, Ethan YB, Penny G, et al. A contemporary analysis of medicolegal issues in obstetric anesthesia between 2005 and 2015. Anesthesia and Analgesia 2019;128:1199–1207. 31094788 10.1213/ANE.0000000000003395

[21] Nagaya K, Fetters DM, Ishikawa M, et al. Causes of maternal mortality in Japan. JAMA 2000;283(20):2661–2667. 10819948 10.1001/jama.283.20.2661

[22] Guglielminotti J, Landau R, Daw J, et al. Association of labor neuraxial analgesia with maternal blood transfusion. Anesthesiology 2023;139(6):734–745. 37585507 10.1097/ALN.0000000000004743 PMC10841247

[23] Kearns J, Kyzayeva A, Halliday L, et al. Epidural analgesia during labor and severe maternal morbidity: Population-based study. BMJ 2024;385:e077190. 38777357 10.1136/bmj-2023-077190 PMC11109902

